# Neuropathic pain drives anxiety behavior in mice, results consistent with anxiety levels in diabetic neuropathy patients

**DOI:** 10.1097/PR9.0000000000000651

**Published:** 2018-05-24

**Authors:** Christine B. Sieberg, Caitlin Taras, Aya Gomaa, Chelsea Nickerson, Cindy Wong, Catherine Ward, Georgios Baskozos, David L.H. Bennett, Juan D. Ramirez, Andreas C. Themistocleous, Andrew S.C. Rice, Pallai R. Shillo, Solomon Tesfaye, Robert R. Edwards, Nick A. Andrews, Charles Berde, Michael Costigan

**Affiliations:** a Department of Anesthesiology, Perioperative and Pain Medicine, Boston Children's Hospital, Boston, MA, USA; b Department of Psychiatry, Harvard Medical School, Boston, MA, USA; c Biobehavioral Pediatric Pain Lab, Boston Children's Hospital, Boston, MA, USA; d Department of Neurobiology, Kirby Neurobiology Center, Boston Children's Hospital, Harvard Medical School, Boston, MA, USA; e Nuffield Department of Clinical Neurosciences, University of Oxford, Oxford, United Kingdom; f Brain Function Research Group, School of Physiology, Faculty of Health Sciences, University of the Witwatersrand, Johannesburg, South Africa; g Pain Research Group, Department of Surgery and Cancer, Faculty of Medicine, Imperial College London, Chelsea and Westminster Hospital Campus, London, United Kingdom; h Diabetes Research Unit, Sheffield Teaching Hospitals NHS Foundation Trust, Sheffield, United Kingdom; i Department of Anesthesiology, Brigham and Women's Hospital, Harvard Medical School, Boston, MA, USA

**Keywords:** Pain, Anxiety, Stress, Spared nerve injury, Neuropathy, Sex differences

## Abstract

**Background:**

Epidemiological studies in patients with neuropathic pain demonstrate a strong association with psychiatric conditions such as anxiety; however, the precipitating pathology between these symptoms remains unclear. To investigate this, we studied the effects of lifelong stress on levels of neuropathic pain–like behavior and conversely, the effects of chronic neuropathic injury on anxiety-like status in male and female mice. In addition, we assayed this link in painful and painless diabetic peripheral neuropathy patients.

**Methods:**

Male and female mice were subject to ongoing life-stress or control living conditions. Baseline sensitivity and anxiety tests were measured followed by spared nerve injury (SNI) to the sciatic nerve. Subsequent sensory testing occurred until 3 weeks after SNI followed by anxiety tests between 4 and 6 weeks after SNI.

**Results:**

Levels of tactile or cold allodynia did not differ between adult mice subject to lifelong chronic stress, relative to nonstressed controls, for at least 3 weeks after SNI. By contrast, longer-term neuropathic mice of both sexes displayed pronounced anxiety-like behavior, regardless of exposure to stress. If sex differences were present, females usually exhibited more pronounced anxiety-like behavior. These ongoing anxiety behaviors were corroborated with plasma corticosterone levels in distinct animal groups. In addition, data from patients with painful and nonpainful diabetic neuropathy showed a clear relationship between ongoing pain and anxiety, with females generally more affected than males.

**Discussion:**

Taken together, these data demonstrate a strong link between chronic neuropathic pain and chronic anxiety, with the driver of this comorbidity being neuropathic pain as opposed to on-going stress.

## Abstract 2

Supplemental Digital Content is Available in the Text.

## 1. Introduction

Studies show a strong comorbidity of chronic neuropathic pain with anxiety disorders and depression.^21,33^ These associations are also present in young patients,^18,37^ which is concerning because of the potential impact on neural development during critical stages of development, with long-term consequences in adulthood.^5,41^ The strong links between ongoing pain and chronic anxiety are well known, but it remains difficult to separate cause and effect in these 2 highly correlated diseases.^12,34^ How biological sex interacts with these disorders is also not well understood, although both chronic anxiety and pain conditions are more prevalent in females.^20^ In addition to determining the etiology of chronic pain and anxiety, understanding their relationship may help target treatments more effectively, for instance, in determining which patients with chronic pain are likely to benefit from drugs with analgesic and anxiolytic properties, for example, gabapentin or duloxetine.^13^

As there is a need to develop a model that allows for insight into the affective component of chronic pain, the aim of this study, which includes preclinical and clinical data, was to characterize the nature of the link between chronic neuropathic pain and ongoing anxiety-like behavior.

## 2. Methods

### 2.1. Animals

All animal procedures were approved by the Boston Children's Hospital Animal Care and Use Committee, under animal protocol numbers 15-04-2928R and 16-01-3080R. All experiments were conducted in a blinded fashion in a quiet room from 09:00 to 18:00. Male and female adult C57BL/6 mice (delivered 6–7 weeks for use at 9 weeks) were obtained from Charles River Labs (CRL). Time-mated female mice were delivered 1 week before birth from CRL. Special care was taken to not disturb the mice (eg, cage changing) on test days, to avoid transient stress confounds. Mice were housed with their littermates (up to 5 mice per cage) in OptiMICE cages with food and water ad libitum (temperature 22 ± 1°C, 50% relative humidity, lights on from 07:00 to 19:00).

Each behavioral test was performed on 2 independent cohorts of male and female mice, the data checked for consistency then merged.

### 2.2. Treatment timeline

**Figure 1.**
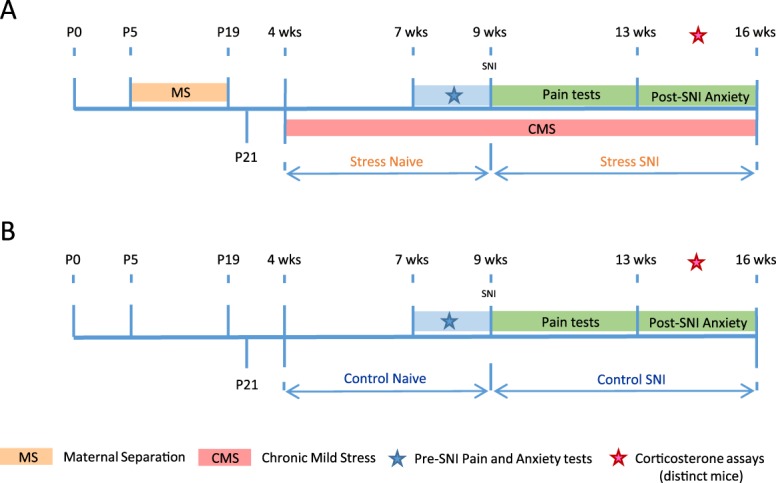
Experimental timeline for male and female mice differentially subjected to ongoing life stress (A) or control unstressed mice (B) before SNI. Baseline sensitivity tests were measured before SNI and anxiety tests were performed before and after SNI within the time frames shown. A separate group of animals were used for plasma blood draws and these assays were measured between 4 and 5 weeks after SNI and within the same time frame for control and ongoing life-stress animals. SNI, spared nerve injury.

Figure 1 shows the timeline for mice that were exposed to ongoing life stress or left alone, before spared nerve injury (SNI)-induced neuropathic pain. Baseline sensitivity tests were measured before SNI injury (in 7–8-week-old mice) and at defined points following it. With the exception of 2 weeks in early life, chronically stressed animals were continuously subject to maternal separation (MS) or chronic mild stress (CMS) throughout the experiment. Plasma corticosterone levels were measured in 3 distinct groups of mice, naive (nonstressed); mice with SNI; or mice that underwent MS followed by CMS. Each was age matched to the SNI results and taken at 4 to 5 weeks after nerve injury.

### 2.3. Chronic stressors

#### 2.3.1. Maternal separation

From P5, pups were separated from their mother's home cage and placed on a warm surface (30°C) for 2 hours between 11:00 and 13:00 each day and then returned to their home cage dams. This procedure was repeated daily until P19. At P21, the mice were separated by sex and housed with their littermates up to n = 5 per cage. After this, the mice were left for 1 week until P29 (4 weeks).

#### 2.3.2. Chronic mild stress

Previously, maternally separated mice were subject to CMS from 4 weeks of age. One of 8 stressors was randomly performed on each cage of animals every day for 6 of the 7 days in the week based on previous studies.^27,44^ The stressors were: (1) cage shake (horizontal shaking, 5 minutes); (2) cold swim (5 minutes per mouse); (3) cage tilt (45° for 8 hours); (4) space reduction (50% cage space for 8 hours); (5) moist bedding (400 mL of water tipped onto bedding and mice left for 8 hours); (6) overnight food deprivation (16 hours); (7) daytime water deprivation (8 hours); and (8) overnight illumination (lights on from 19:00 to 07:00 in addition to the normal day light period). Once the list of 8 interventions was complete, a new random run of the 8 different interventions was performed and this procedure was repeated until the end of the experiment.

### 2.4. Peripheral nerve injury

Mice were anesthetized with isoflurane (2%–4%) at 9 weeks and SNI surgery performed; the tibial and common peroneal branches of the sciatic nerve were tightly ligated with a silk suture and transected distally, whereas the sural nerve was left intact.^4,8^

### 2.5. Sensitivity assays

#### 2.5.1. Measures of mechanical and cold allodynia

The behavioral time course of tactile and cold sensitivity before and after SNI in C57BL/6 mice was measured as in Refs. 4,8. Mice were tested twice at baseline and then 7, 14, and 21 days after SNI. For the CMS cohort, the mice were not subject to the CMS procedure on the day of testing. Mechanical allodynia was measured using von Frey filaments (Touch-Test Sensory Evaluators; North Coast Medical, Inc, Gilroy, CA) ranging from 0.02, 0.04, 0.07, 0.16, 0.4, 0.6, 1, and 2 g, respectively. Each filament was tested 10 times in increasing order starting with the filament producing the lowest force. Von Frey filaments were applied at least 3 seconds after the mice had returned to their initial resting state. For the baseline mechanical sensitivity test, all filaments were applied and the number of withdrawals was recorded. For tactile allodynia, the minimal force filament for which animals presented either a brisk paw withdrawal and/or an escape attempt in response to at least 5 of the 10 stimulations determined the mechanical response threshold. Cold allodynia was assayed by applying a 5 μL drop of acetone to the hind paw, ipsilateral to SNI injury, and measuring the amount of time the animal spent flinching/licking/biting the paw in seconds.

### 2.6. Anxiety assays

#### 2.6.1. Elevated plus maze

The elevated plus maze consisted of 2 open and 2 closed arms each opposite its counterpart, extended from a central platform to create a plus shape elevated 50cm above the floor. Mice were placed on the center platform of the maze, facing a closed arm, and allowed to explore the apparatus for 5 minutes. The percent of time spent in open arms was used as a surrogate measure of anxiety-like states.^30^ Distance travelled in the closed arms was considered as a measure of locomotor activity.

#### 2.6.2. Holeboard

The apparatus consisted of a square arena surrounded by a wall. Sunken into the arena floor were 9 holes equally spaced in 3 rows. Activity (horizontal movement and investigation of the holes) was detected by infrared beams and recorded by a computer. The test duration was 15 minutes and was initiated by placing a mouse into the periphery of the arena. Total distance traveled in the periphery (ambulation), total time spent in the center (nonanxious behavior), and total number of nose pokes into the holes (exploratory behavior) were assessed.

#### 2.6.3. Light dark box

The apparatus consisted of a 2-compartment chamber connected by a small aperture that allowed free access to both sides. The test began by placing a mouse in the dark compartment and recording behavior; time spent in the lit compartment (nonanxious behavior) and distance traveled in the light zone (ambulation) were recorded.

### 2.7. Determination of plasma corticosterone concentration

Plasma corticosterone levels were measured in 3 distinct groups of mice, naive (nonstressed); mice with SNI; or mice that underwent MS followed by CMS. Each of these groups was further divided into mice that underwent acute restraint stress or remained unstressed in the home cage before blood collection. Restraint stress consisted of mice inserted into 50 mL conical polypropylene tubes for 5 minutes. The tube had holes placed along its length and the conical face removed to aid breathing. After restraint, the mice were returned to their home cage for 20 minutes and then euthanized with carbon dioxide inhalation before cardiac puncture to collect blood samples, stored in 3 mL EDTA tubes. Unrestrained mice were left in their home cage undisturbed before blood collection. All mice were euthanized in a room separated from that of housing before sample collection. All samples were collected between 11:00 and 13:00. Plasma was prepared from the blood samples and stored at −80°C until required. CORT was quantified by ELISA (#ADI-900-097; Enzo Life Sciences, Farmingdale, NY) according to the manufacturer's instructions.

### 2.8. Neuropathic patients

Data are presented on 176 adult patients with diabetes mellitus who participated in the *Pain in Neuropathy Study* (PiNS), a cross-sectional observational multicenter study in the United Kingdom designed to determine the somatosensory phenotype of painful and painless diabetic peripheral neuropathy. The total cohort consists of 191 participants; however, 15 did not complete the anxiety questionnaire. Information on study methodology and sample has been published.^39^

Participants underwent a neurological examination, quantitative sensory testing, nerve conduction studies, and skin biopsy for intraepidermal nerve fiber density assessment. Toronto Clinical Scoring System (TCSS) was used as a screening tool for diabetic peripheral neuropathy and correlates with diabetic neuropathy severity.^6^ Participants also completed a series of questionnaires assessing the presence of pain, pain intensity, pain distribution, and psychological and functional impact of pain. The Depression Anxiety Positive Outlook (DAPOS) measure^31^ was used to assess anxiety; a 7-day pain diary to assess ongoing neuropathic pain intensity^39^; TCSS as a measure of diabetic neuropathy severity; HbA1c (glycated haemoglobin-A1c concentration as a measure of average plasma glucose concentration over the preceding 120 days); age; and body mass index (BMI).

Sample size was determined according to the warm detection threshold for patients with diabetes (Shun et al., 2004), which revealed that a minimum sample size of 34 was required per group for a power of >0.8, SPSS Statistics Version 22 (IBM). Data were tested for normality with the D'Agostino-Pearson normality test and by visual inspection. Data were not normally distributed and are reported as median with interquartile range. Data were compared across multiple groups with the Kruskal–Wallis test (the Dunn post hoc test). In the case of comparisons between 2 groups only, the nonparametric Wilcoxon rank-sum test was used. Spearman correlation analyses were performed to explore associations between DAPOS anxiety and pain intensity. A multivariate analysis was performed to control for potential confounding factors. Ranked nonparametric multivariate analysis of variance (ANOVA) with the Wilk's Lambda statistic and multivariate principal component analysis were performed. All values were normalized and scaled before analysis. Statistical significance set at *P* < 0.05.

## 3. Results

### 3.1. Tactile and cold allodynia in stressed and unstressed mice

**Figure 2.**
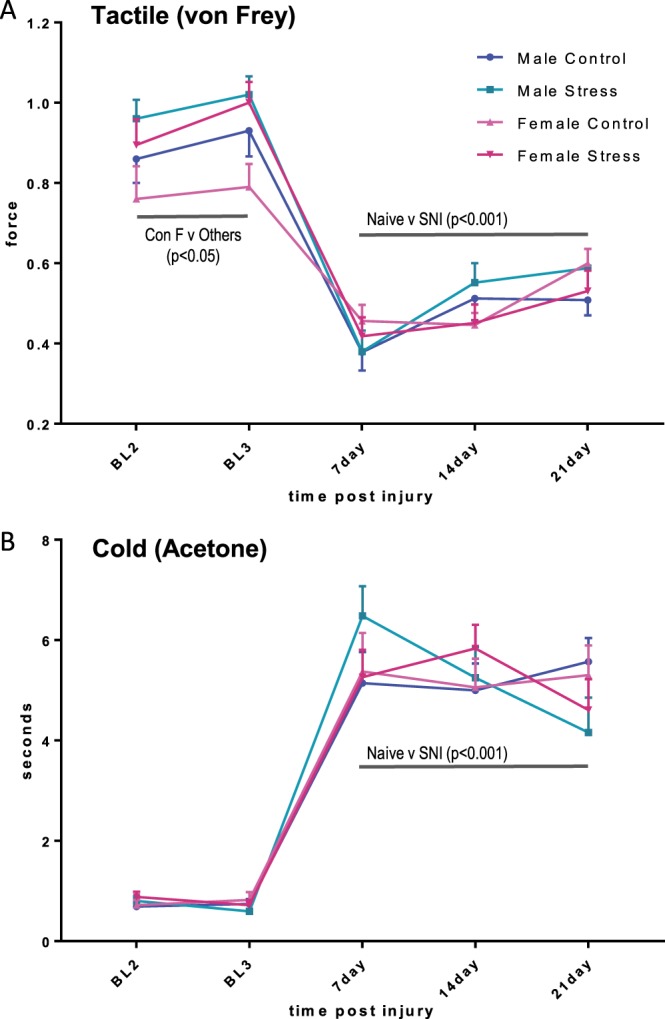
Male and female C57BL/6 chronic stress or nonstressed mice were tested for tactile (A) or cold (B) sensitivity pre- (BL2 and BL3 2 independent baseline values) and post-SNI (each group contained n = 19 to 20 mice). (A) Repeated-measures ANOVA results for tactile allodynia in male and female control and stressed mice demonstrated a main effect for time (*P* < 0.001) indicating a significant effects of SNI over the time course for all mice, and a significant interaction effect (*P* < 0.05). Post-hoc Tukey tests follow-up analysis revealed a significant difference between naive control females and the other groups before surgery. After SNI, all mice, stressed and nonstressed of both sexes, developed strong levels of tactile and cold allodynia by 7 days after injury and this was maintained until at least 21 days. (B) Repeated-measures ANOVA results for cold allodynia in each group demonstrated a main effect for time (*P* < 0.001) indicating a significant effects of SNI over the time course for all mice. Error SEM. ANOVA, analysis of variance; SNI, spared nerve injury.

To measure tactile sensitivity, we used von Frey filaments (Fig. 2A). Baseline means were averaged. The 2 (Sex) × 2 (Group) × 4 (Time) ANOVA revealed a significant main effect of Time [F(3,73) = 86.2, *P* < 0.001], and Group × Time [F(3,73) = 2.6, *P* = 0.05] as well as Sex × Time [F(3,73) = 2.9, *P* < 0.05] interactions. Follow-up univariate analyses indicated that naive (unstressed) female mice showed significantly lower values (greater tactile sensitivity) compared with the other 3 groups at baseline. No group differences were evident thereafter. Collectively, after SNI, all mice developed strong levels of tactile allodynia by 7 days after injury and this was maintained until at least 21 days.

Similarly, cold allodynia (Fig. 2B) developed fully by 7 days after SNI and was sustained for 21 days. The 2 (Sex) × 2 (Group) × 4 (Time) ANOVA revealed a significant main effect of Time [F(3,73) = 125.5, *P* < 0.001], and a significant Group × Time interaction [F(3,73) = 4.7, *P* < 0.01], although follow-up univariate analyses indicated no significant differences from baseline to day 21. Overall, the effect of SNI on tactile and cold hypersensitivity in mice was independent of sex or previous chronic stress.

### 3.2. Long-term neuropathic injury induces anxiety-like behavior in mice (elevated plus maze)

**Figure 3.**
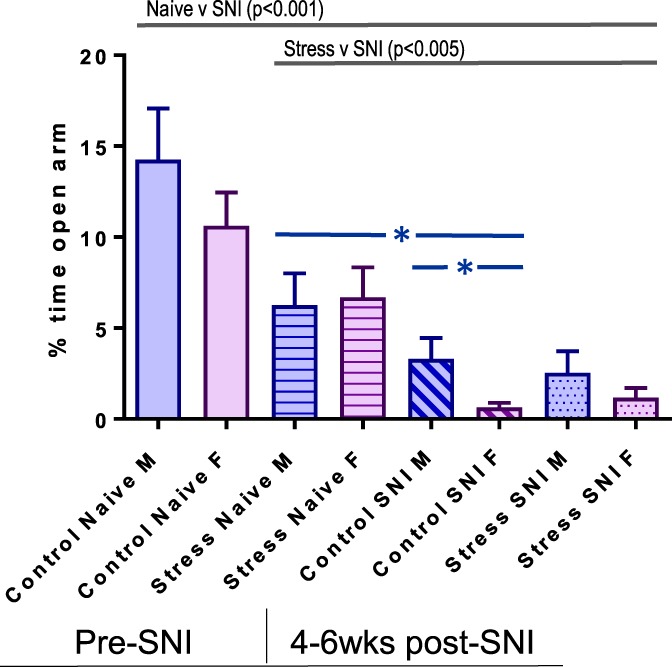
Time spent on the open arms of the elevated plus maze as a measure of anxious-like behavior in male and female C57BL/6 naive, chronic stress, nonstressed post-SNI and chronic stress post-SNI mice (each group contained n = 16–20 mice). Repeated-measures ANOVA results a main effect of SNI (*P* < 0.001), a significant Stress vs SNI interaction (*P* < 0.005), and follow-up Tukey test analyses indicate that the control stress groups spent more time on the open arms control SNI groups (*P* < 0.05, in blue). Error SEM. ANOVA, analysis of variance; SNI, spared nerve injury.

The 2 (Sex: M vs F) × 2 (Group: Control vs Stress) × 2 (Time: N vs SNI) ANOVA (Fig. 3) revealed a significant main effect of Time (SNI) [F(1,69) = 65.4, *P* < 0.001] and a significant Group (Stress) × Time (SNI) interaction [F(1,69) = 9.8, *P* < 0.005], indicating that SNI induces increased anxiety behavior. The chronically stressed groups spent less time on the open arms before SNI (*P* < 0.05), and control female mice spent significantly less time in the open arms after SNI (*P* < 0.05). It is important to note that adding distance traveled (not shown) as a covariate to the ANOVA did not change this pattern of results, suggesting that the observed effects are not a consequence of a general loss of activity levels after SNI.

### 3.3. Long-term neuropathic injury induces anxiety-like behavior in mice (holeboard)

**Figure 4.**
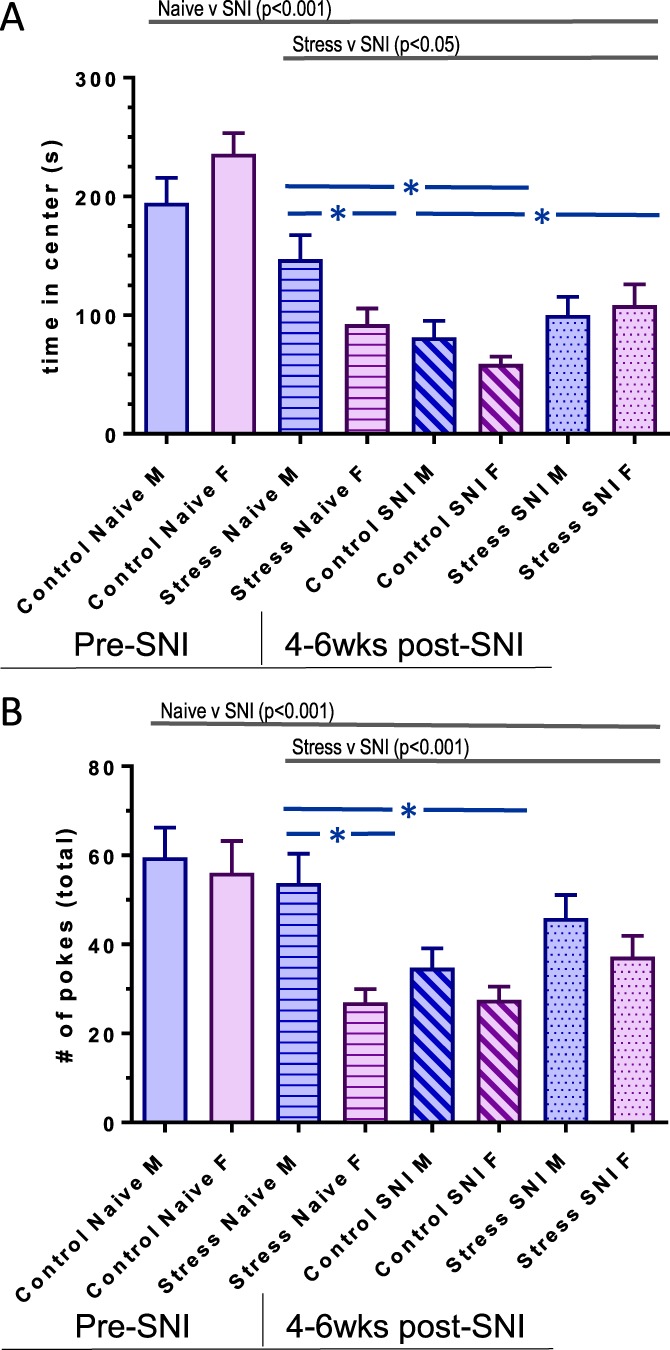
(A) Time spent the center of the holeboard arena as a measure of anxious-like behavior in male and female C57BL/6 naive, chronic stress, nonstressed post-SNI, and chronic stress post-SNI mice (each group contained n = 16–20 mice). Repeated-measures ANOVA results demonstrate a main effect of SNI (*P* < 0.001) with a significant Stress vs SNI interaction (*P* < 0.05). Follow-up analyses revealed that chronic stress mice spent significantly more time in the center of the holeboard than post-SNI control mice (*P* < 0.05, in blue). Male chronically stressed mice spent significantly more time in the center than their female counterparts (*P* < 0.05, in blue). Leading to a significant Sex × Stress × SNI interaction (*P* < 0.005, not shown on graph). (B) The number of nose pokes into all the holes in the holeboard as a measure of exploratory behavior. Repeated-measures ANOVA results demonstrate a main effect of SNI (*P* < 0.001), main effect of Stress (*P* < 0.001) and a main effect of Sex (*P* < 0.005, not shown on graph). Post-hoc analysis revealed that stressed female mice engage in fewer holeboard nose pokes than males (*P* < 0.05, in blue) and stressed mice are more exploratory than control SNI mice (*P* < 0.05, in blue). Error SEM. ANOVA, analysis of variance; SNI, spared nerve injury.

In the holeboard arena (Fig. 4), the 2 (Sex) × 2 (Group) × 2 (Time) ANOVA on the index of time spent in the holeboard center (Fig. 4A) revealed significant main effects of Time (SNI) [F(1,67) = 68.4, *P* < 0.001], Group (Stress) [F(1,67) = 5.4, *P* < 0.05], a significant Group × Time interaction [F(1,69) = 44.6, *P* < 0.001], and a significant Group × Sex × Time interaction [F(1,67) = 9.3, *P* < 0.005]. Follow-up analyses indicated that the chronically stressed groups spent less time in the center than the unstressed groups (*P* < 0.05), and sex differences were only evident in the chronically stressed group (with stressed females demonstrating less center time than stressed males, *P* < 0.05). After SNI, only the unstressed groups showed significant reductions in time spent in the holeboard center; indeed, chronically stressed females had higher scores than unstressed females after SNI (*P* = 0.01). These findings suggest that chronic stress amplifies anxiety behaviors, particularly in females, but that chronic stress also seems to buffer the anxiety-promoting impact of SNI. Adding distance traveled in the periphery as a covariate did not change the pattern of group differences (not shown).

The 2 (Sex) × 2 (Group) × 2 (Time) ANOVA for number of holeboard nose pokes (exploratory behavior) (Fig. 4B) revealed a significant main effect of Time (SNI) [F(1,67) = 13.9, *P* < 0.001], a significant main effect of Sex [F(1,67) = 7.5, *P* < 0.005], and a significant Group × Time interaction [F(1,69) = 16.5, *P* < 0.001]. After SNI, nose pokes decreased in all groups, with no significant sex or group differences. Adding distance traveled as a covariate did not change the pattern of results.^32^

### 3.4. Long-term neuropathic injury induces anxiety-like behavior in mice (light dark box test)

**Figure 5.**
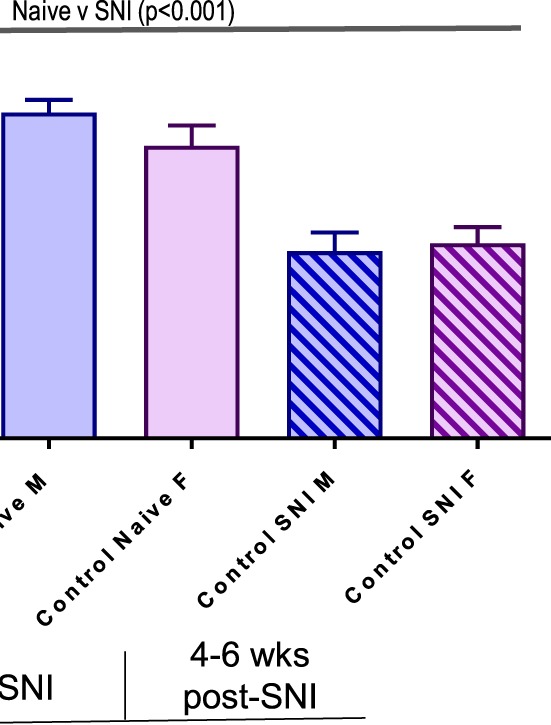
Time spent in the light area of the light dark box as a measure of anxious-like behavior in male and female C57BL/6 naive, nonstressed post-SNI mice (each group contained n = 16–20 mice). Mice of both sexes spent significantly less time in the light arena after SNI (*P* < 0.001). Error SEM. SNI, spared nerve injury.

The 2 (Sex) × 2 (Time) ANOVA for time spent in the light during the light dark box assay (Fig. 5) revealed a significant main effect of Time (SNI) [F(1,33) = 57.7, *P* < 0.001], with no main effect of Sex and no significant interaction (Fig. 5). Both male and female mice exhibited significant reductions in time spent in the light after SNI. These effects were not attributable to reductions in locomotion; adding distance as a covariate did not change the pattern of results.

### 3.5. Plasma corticosterone levels increase due to spared nerve injury and chronic stress

Given the behavioral findings that long-term SNI drives anxiety-like behavior, we compared plasma corticosterone levels in mice at 4 to 5 weeks after SNI to naive unstressed controls and chronically stressed mice. We also assayed corticosterone levels in restrained and unrestrained mice (see Methods), with unrestrained levels of corticosterone being a marker of baseline stress levels. Over time, chronically increased plasma corticosterone leads to the desensitization of the glucocorticoid receptor system, resulting in larger levels of corticosterone produced in reaction to a novel acute stress.^24^

**Figure 6.**
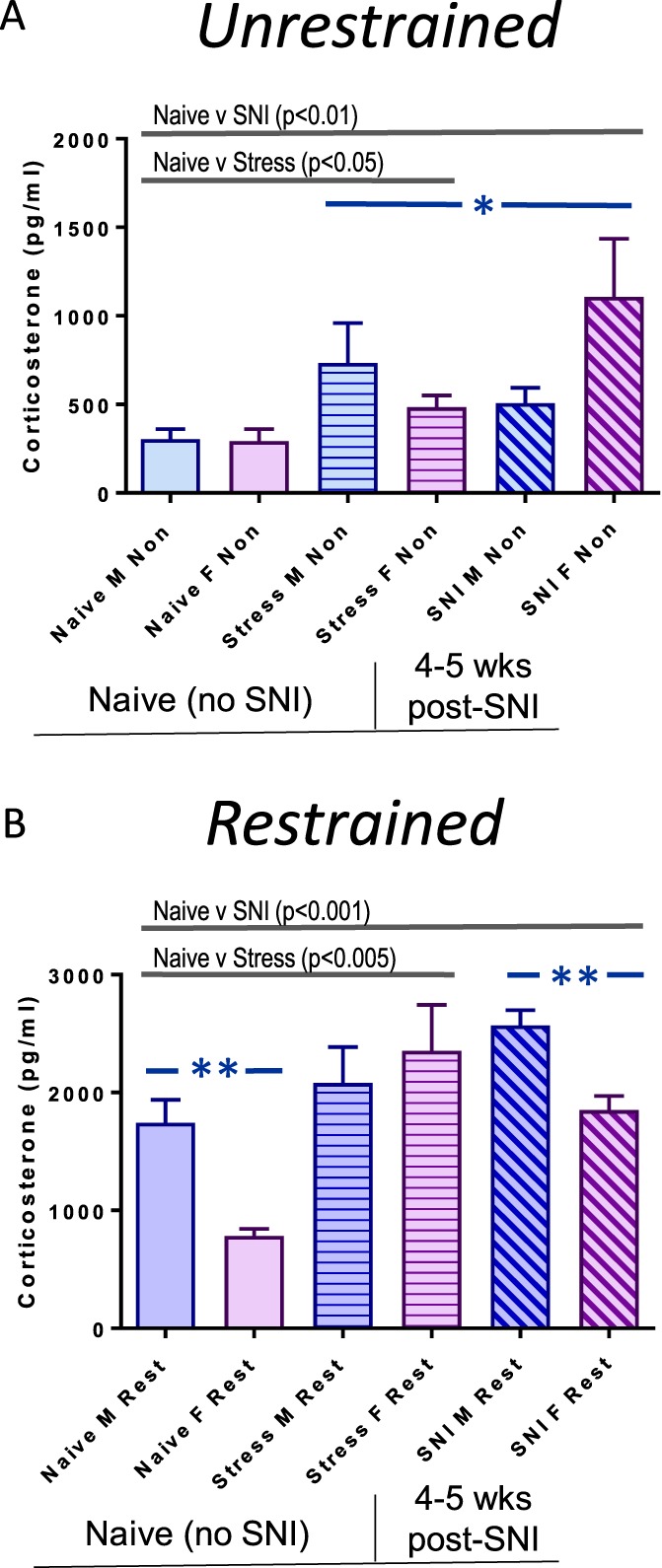
Plasma corticosterone levels in unrestrained (A) and restrained (B) male and female, control and stressed mice before and after SNI (each group contained n = 6–10 mice). (A) Within the unrestrained group, repeated-measures ANOVA demonstrates a main effect for SNI (*P* < 0.01) and for Stress (*P* < 0.05) with a significant difference between these groups (*P* < 0.05). (B) Within the restrained group, repeated-measures ANOVA demonstrates a main effect of SNI (*P* < 0.001) and for stress (*P* < 0.005). Post-hoc analysis demonstrates that a significant effect of sex (*P* < 0.01) was revealed in naive and post-SNI mice, with higher levels of plasma corticosterone in males relative to females. Error SEM. ANOVA, analysis of variance; SNI, spared nerve injury.

As expected, unrestrained animals produced significantly less corticosterone than the restrained animals (*P* < 0.001) across all subgroups. In the unrestrained group (Fig. 6A), the naive and chronic stress groups differ at *P* < 0.05 and the naive and SNI groups differ at *P* < 0.01, with the chronic stress and SNI groups differing at *P* < 0.05, with no sex differences. Long-term SNI is therefore a stronger inducer of basal corticosterone levels than lifelong chronic stress. In the restrained group (Fig. 6B), the naive vs the chronic stress comparison is significant at *P* < 0.005 with the naive vs SNI significant at *P* < 0.001, suggesting that chronic stress and SNI are each capable of significantly increasing corticosterone levels. Control males and post-SNI males had higher corticosterone levels than control and post-SNI females, respectively (*P* < 0.01).

Overall, regardless of restraint or nonrestraint status, long-term SNI and lifelong chronic stress result in greater levels of plasma corticosterone production than that seen in naive animals, consistent with these treatments being at least equally effective stressors.

### 3.6. Anxiety scores in patients with painful vs nonpainful diabetic neuropathy

**Figure 7.**
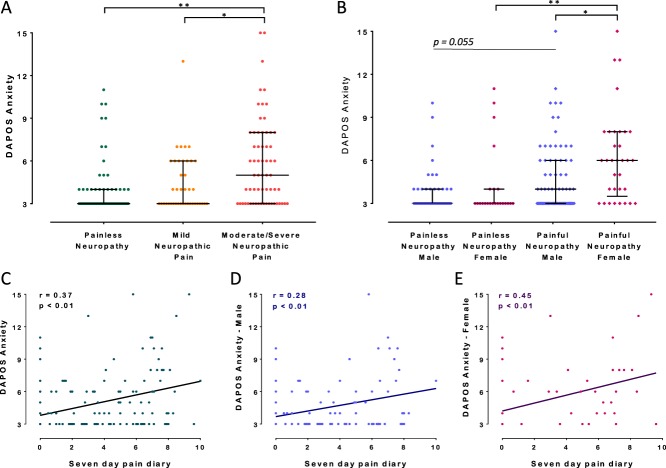
(A) Scatter plot and median (interquartile range) of DAPOS Anxiety scores for study participants with painless and painful diabetic neuropathy. Study participants with painful diabetic neuropathy were subdivided into those with mild and moderate/severe neuropathic pain based on the mean pain scores obtained from the 7-day pain intensity pain diary (0–3.9 mild neuropathic pain, and 4–10 moderate/severe neuropathic pain). (B) Scatter plot and median (interquartile range) of DAPOS Anxiety scores for male and female study participants with painless and painful diabetic neuropathy. The Kruskal–Wallis and the Dunn multiple comparison test: *P < 0.05, **P < 0.01. (C–E) Scatter plot of DAPOS Anxiety scores against 7-day pain intensity diary mean in all study participants with diabetic neuropathy (C). Male study participants with diabetic neuropathy (D) and female study participants with diabetic neuropathy (E). DAPOS Anxiety scores were correlated with 7-day pain intensity mean for all study participants with female study participants showing a greater correlation than male study participants. DAPOS, Depression Anxiety Positive Outlook.

In the PiNS study cohort, anxiety scores increased successively as levels of neuropathic pain increased, irrespective of sex (Fig. 7A). Anxiety scores were higher for participants with painful neuropathy compared with same-sex study participants with painless neuropathy (Fig. 7B). Female participants with painful neuropathy reported significantly higher anxiety scores compared with male participants with painful neuropathy (*P* < 0.05), with no sex differences for painless neuropathy (Fig. 7B).

**Figure 8.**
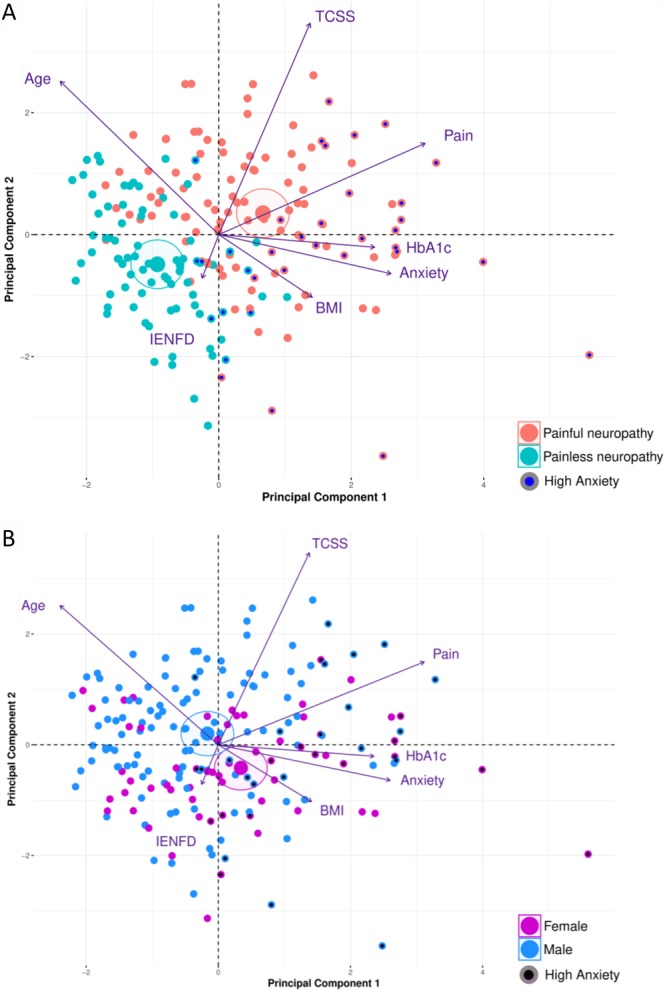
Multivariate PCA of patients with painful and painless neuropathy. Variables: age; body mass index (BMI); glycated haemoglobin-A1c concentration (HbA1c); intraepidermal nerve fiber density (IENFD); DAPOS Anxiety score (Anxiety); extent of peripheral nerve damage (TCSS), and Pain Diary 7-day mean (chronic pain) were normalized and used to construct principal components. (A) Painful/painless neuropathy patient diagnosis was not used to construct the PC plots but was tested for differences in principal components using 1-way ANOVA. PC1 was strongly characterized painful neuropathy patients (estimate: 0.79, *P* value: 3.89e−19) with PC2 also significantly characterized by patients with painful neuropathy (estimate: 0.42, *P* value: 1.87e−07). Individuals factor map based on the 2 first principal components. Minor dots represent individual patients, diagnosed with painful (red) or painless neuropathy (green). High anxiety patients (DAPOS Anxiety score ≥ third quantile threshold) are indicated by dark center point. Large dots and ellipses represent the euclidean centroids of painful and painless neuropathy patient groups. PC1 is characterized and correlated with chronic pain, anxiety, HbA1c concentration and BMI and anticorrelated with age. Of which, chronic pain (correlation coef. = 0.74, *P* value = 9.54e−35) and anxiety (correlation coef. = 0.64, *P* value = 9.36e−24) are the most important factors. PC2 is characterized and correlated with TCSS, age, and chronic pain and anticorrelated with BMI and IENFD. Of which, TCSS (correlation coef. = 0.82, *P* value = 5.13e−49) and age (correlation coef. = 0.60, *P* value = 5.61e−20) are the most important factors. Only variables with significant correlations are plotted (*P* value <0.05), arrow length is proportional to Pearson correlation coefficient. (B) Patient sex was not used to construct the principal components' PC plots but was tested for differences in principal components using 1-way ANOVA. PC1 was characterized slightly by females (estimate: 0.25, *P* value: 1.42e−02). PC2 was modestly characterized by males (estimate: 0.30, *P*-value: 3.43e−04). Minor dots represent individual patient's sex with male (blue) and female (purple), center points designate high anxiety patients. Large dots and ellipses represent the euclidean centroids of male and female patient groups. ANOVA, analysis of variance; DAPOS, Depression Anxiety Positive Outlook; PCA, principal component analysis; TCSS, Toronto Clinical Scoring System.

Scatter plots of DAPOS Anxiety and pain intensity showed a significant correlation *r* = 0.37, *P* < 0.01 (Fig. 7C), which was strongest for female study participants *r* = 0.45, *P* < 0.01 (Fig. 7E). A multivariate analysis was performed to control for potential confounding factors (Fig. 8 and Supplemental Fig. 1, available online at http://links.lww.com/PR9/A18). Multivariate principal component analysis included: Sex, age, BMI, HbA1c, intraepidermal nerve fiber density, DAPOS, anxiety, TCSS, and neuropathic pain intensity. Variables were normalized and used to construct principal components. Multivariate analysis effectively separated participants diagnosed with painful and painless neuropathy. Painful neuropathy participants are better associated with principal component 1 (PC1) (estimate = 0.79, *P* = 3.8e−19) than PC2 (estimate = 0.41, *P* = 1.87e−07), see centroids of patient groups and color coding of individual pain patients (Fig. 8A). PC1 is strongly correlated with neuropathic pain intensity (cor = 0.74, *P* = 9.54e−35), anxiety (cor = 0.64, *P* = 9.36e−24), and HbA1c (cor = 0.57, *P* value = 3.38e−18). Furthermore, BMI showed a modest correlation (cor = 0.33, *P* = 1.61e−06) and age was anticorrelated (cor = −0.57, *P* = 6.71e−18). PC2 is strongly correlated with TCSS (cor = 0.82, *P* = 5.13e−49) and age (cor = 0.59, *P* = 5.61e−20) (Fig. 8A). Female participants are associated with PC1 (estimate = 0.25, *P* = 1.42e−02), whereas male participants are associated with PC2 (estimate = 0.30, *P* = 3.43e−04), although both associations are relatively modest (Fig. 8B). The nonparametric Wilcoxon rank-sum test showed that anxiety scores (*P* = 0.01), TCSS (*P* = 0.0079), BMI (*P* = 9.9e−05), age (*P* = 0.014), and daily pain intensity (*P* = 0.043) were significantly different between the sexes (Supplemental Fig. 1, available online at http://links.lww.com/PR9/A18).

In summary, multivariate analysis found significant relationships between anxiety scores and multiple variables including painful neuropathy diagnosis, neuropathic pain intensity, plasma glucose concentration, and BMI. Study participants with more severe neuropathic pain reported greater levels of anxiety. Women reported higher anxiety scores than males, although levels of anxiety for both sexes with painful neuropathy were significantly increased.

## 4. Discussion

Preclinical and clinical data confirm the substantial comorbidity between chronic pain and affective distress;^16,33,37^ however, it is less clear whether psychiatric comorbidities pre-date or result from the onset of neuropathic pain. Current literature on this issue are conflicted; although some studies indicate that early-life stress exacerbates allodynia,^7,26,28^ others suggest that chronic stress reduces levels of neuropathic mechanical allodynia.^14,36^ Our data suggest the underlying mechanisms of nerve injury–induced tactile or cold allodynia are not modulated by ongoing stress or sex, at least until 3 weeks after SNI in C57BL/6 mice.

Next, we assayed longer-term neuropathic mice (4–6 weeks after SNI) using 3 well-characterized tests of neophobia. Again, published data in this area are contradictory, with some showing no association between nerve injury and anxiety behavior,^17,40^ whereas others show that neuropathic injury increases anxiety-like behavior.^9,23,25,38,45^ Interestingly, our findings combined with these other studies suggest a time sensitivity to these events. Within the first few weeks after nerve injury, affective state was relatively unaffected,^17,40^ but with neuropathic pain for more than 4 weeks after injury, anxiety-like behavior becomes apparent.^9,23,25,38,45^ Defining ongoing long-term pain hypersensitivity as the precipitant of clinical anxiety is an essential improvement in our understanding of these 2 strongly linked conditions.

We also assayed the effects of sex, and where differences were present, females were usually more susceptible to anxiety-like behaviors, despite similar levels of ongoing tactile and cold hypersensitivities after SNI. A limitation here is that the stage of the estrous cycle was not assessed in the female animals and further work is needed to look at the different stages of the cycle in the context of the pain and anxiety relationships we have uncovered to determine the impact on the estrous cycle.

We also found that chronic lifelong stress, as well as long-term SNI, increased tonic levels of plasma corticosterone in both sexes. Increased regulation of corticosterone was still evident after acute stress relative to naive mice, suggesting chronic desensitization of corticosterone signaling by lifelong stress or long-term SNI. Other published data also demonstrate increased plasma corticosterone levels after nerve injury,^3,22,42^ or chronic stress,^10,19,35^ as well as evidence for upregulation of corticosterone signaling pathways after peripheral nerve injury.^42,43^ Overall, our data demonstrate that long-term SNI and chronic stress are about equivalent in increasing plasma corticosterone levels, suggesting similar levels of signaling through this endogenous stress system for each condition.

Although chronic neuropathic pain and ongoing anxiety have been linked, the fact that neuropathic injury precipitates anxiety-like behavior has not been defined and the timing of its appearance has not been clear. Our data also offer mechanistic evidence as to why many anxiolytic drugs, such as gabapentin/pregabalin, tricyclic antidepressants, and serotonin–norepinephrine reuptake inhibitors are also effective neuropathic pain medications.^11,13^ Such drugs can increase norepinephrine in the spinal cord, which alleviates pain^29^; however given our data, it is possible that the reduction of high anxiety-like levels alone also contribute to the efficacy of these drugs. It would be of future interest to test how well anxiolytics with an antineuropathic pain component reduce comorbid anxiety-like behavior levels as well as pain-like behavior scores and in addition analyze how anxiolytic drugs without a recognized antineuropathic effect would perform in the same tests. As in rodents, it has been difficult to establish in humans whether psychological distress is a precursor to^34^ or consequence of^12^ living with chronic pain, so comparing those patients with painful vs painless diabetic neuropathy offers some potential insight. Our patient data are consistent with the animal findings, in that anxiety scores were higher for those with painful neuropathy, and women reported higher levels of anxiety and a stronger pain–anxiety relationship. Our findings align with other diabetic neuropathy studies where greater pain intensity relates to more anxiety and depression.^2,15^ Here, we demonstrate a strong correlation between increased anxiety levels in either mice subject to SNI injury or patients suffering painful diabetic neuropathy, one may postulate that these conditions are too dissimilar to compare; however, we would suggest that the remarkable similarity of risk profiles demonstrated in these data, including a female predominance speaks to an essential mechanism through mammalian biology, one that basically connects longer-term ongoing painful sensory input with central changes that precipitate marked anxiety in mice or patients. These correlations hold true, despite the initial source of chronic painful sensory input, be it metabolic disease to the peripheral nervous system or frank experimental nerve injury and of course across species.

Anxiety-like behaviors in mice subject to long-term SNI were comparable with those in mice subject to both long-term SNI and lifelong stress. Translated to patients, these results would suggest that neuropathic pain and anxiety do not combine to produce greater anxiety, as neuropathic pain fully drives anxiety symptoms. A large-scale study by Attal et al.^1^ was consistent with this view, as anxiety scores^46^ increased successively and significantly from pain-free subjects to subjects with non-neuropathic pain, to subjects with neuropathic pain, who exhibited near-maximal scores. Interestingly, depression was less strongly impacted by pain, suggesting that neuropathic pain symptoms are so unpleasantly salient that they overwhelm other stressors and link predominantly to anxiety within the central nervous system. Our data may therefore be instructive when dealing with neuropathic pain patients with anxiety, suggesting that successfully treating ongoing pain is likely to be the key determinant to removing patients' ongoing anxiety.

## Disclosures

The authors have no conflict of interest to declare.

This study were supported by a Boston Children's Hospital Office of Faculty Development Career Development Grant and NIH grant (K23 GM123372-01) awarded to C.B. Sieberg and the Sara Page Mayo Endowment for Pediatric Pain Research and Treatment, and the Department of Anesthesiology, Perioperative and Pain Medicine at Boston Children's Hospital (CB). The Pain in Neuropathy Study was supported by the Wellcome Trust through a Strategic Award to the London Pain Consortium (ref. no. 083259); research reported in this publication was part of the International Diabetic Neuropathy Consortium (IDNC) research programme, which is supported by a Novo Nordisk Foundation Challenge programme grant (Grant number NNF14SA0006). A.C. Themistocleous is supported by the International Diabetic Neuropathy Consortium (IDNC) research programme, which is supported by a Novo Nordisk Foundation Challenge programme grant. D.L.H. Bennett is a Wellcome Senior Clinical Scientist (202747/Z/16/Z). D.L.H. Bennett, A.S.C. Rice, and A.C. Themistocleous are members of the DOLORisk consortium funded by the European Commission Horizon 2020 (ID633491).

## Appendix A. Supplemental digital content

Supplemental digital content associated with this article can be found online at http://links.lww.com/PR9/A18.
